# Trends in outpatient care utilization for patients with established atrial fibrillation before and after the Covid-19 pandemic: a nationwide analysis of claims data

**DOI:** 10.1186/s13104-025-07569-6

**Published:** 2025-11-27

**Authors:** Lanting Yang, Shangbin Tang, Jingchuan Guo, Nico Gabriel, A. Mark Fendrick, Nimish Patel, Utibe R. Essien, Jared W. Magnani, Walid F. Gellad, Inmaculada Hernandez

**Affiliations:** 1 Division of Clinical PharmacySkaggs School of Pharmacy and Pharmaceutical Sciences, University of California, San Diego, 9500 Gilman Dr, La Jolla, CA 92093 USA; 2 Department of Pharmaceutical Outcomes and Policy, University of Florida College of Pharmacy, Gainesville, FL USA; 3 Department of Internal Medicine, Department of Health Management and Policy, University of Michigan, Ann Arbor, MI USA; 4 Division of General Internal Medicine and Health Services Research, David Geffen School of Medicine, University of California, Los Angeles, CA USA; 5 Center for the Study of Healthcare Innovation, Implementation & Policy, Greater Los Angeles VA Healthcare System, Los Angeles, CA USA; 6 Division of Cardiology, Department of Medicine, University of Pittsburgh School of Medicine, Pittsburgh, PA USA; 7 Division of General Internal Medicine, University of Pittsburgh School of Medicine, Pittsburgh, PA USA

**Keywords:** Atrial fibrillation, Outpatient visits, Covid-19

## Abstract

**Objective:**

To evaluate trends in outpatient visits among Medicare beneficiaries with established AF before and after the pandemic and to investigate differences in outpatient care utilization patterns based on baseline utilization levels.

**Results:**

We analyzed 2018–2022 Medicare data to assess outpatient visit trends among 124,483 beneficiaries with atrial fibrillation (AF), categorizing them into quartiles based on pre-pandemic in-person visit frequency. We found that in-person visits declined while telehealth use increased across all groups during the pandemic. After the pandemic, total visits remained below pre-pandemic levels for higher-utilizing groups after the pandemic.

**Supplementary Information:**

The online version contains supplementary material available at 10.1186/s13104-025-07569-6.

## Introduction

 The COVID-19 pandemic had a major impact on the management of chronic diseases, resulting in a reduction in the rates of in-person visits and a rapid increase in telehealth utilization [1]. Atrial fibrillation (AF) is a particularly relevant disease state to evaluate pandemic disruptions of outpatient care for chronic disease, due to the need for monitoring of heart rate and management of anticoagulation therapy and comorbid conditions [2–5]. 

Previous work documented important decreases in the rates of outpatient visits for patients with AF following the onset of the COVID-19 pandemic; however, it remains unclear when the levels of outpatient visits returned to the pre-pandemic level and to what extent telehealth contributed to closing the gap in outpatient care triggered by the pandemic [6–9]. Additionally, it remains unclear to what extent pandemic disruptions of outpatient care utilization, differed across patient subgroups based on baseline outpatient utilization. This is important because patients with higher levels of utilizations are more vulnerable to disruptions in healthcare delivery, which can lead to adverse outcomes if gaps in care are not addressed.

We used Medicare data from 2018 to 2022 to investigate whether outpatient visits among Medicare beneficiaries with AF returned to pre-pandemic levels by December 2022, to quantify the role of telehealth in closing this gap in care, and to describe how recovery patterns varied based on the baseline outpatient care utilization.

## Methods

We used 2018–2022 claims data from a 5% random sample of fee-for-service Medicare beneficiaries. First, we selected patients who had a diagnosis of AF at any time before March 18, 2019, which was the index date (Supplemental Fig. 1). This specific date was selected as index date because outcomes were assessed at 90-day intervals, and March 18, 2019 marks exactly four 90-day intervals prior to March 11, 2020 - the date when the World Health Organization declared COVID-19 a pandemic [10]. AF was defined following the Centers for Medicaid and Medicare Services Chronic Conditions Data Warehouse definition [11–13]. Second, we excluded patients with a diagnosis of valvular disease in the 12 months prior to the index date. Finally, we constrained sampling to patients with continuous enrollment for 12 months before and after the index date. Patients were followed from index date until December 2022, death, or disenrollment.

**Fig. 1:**
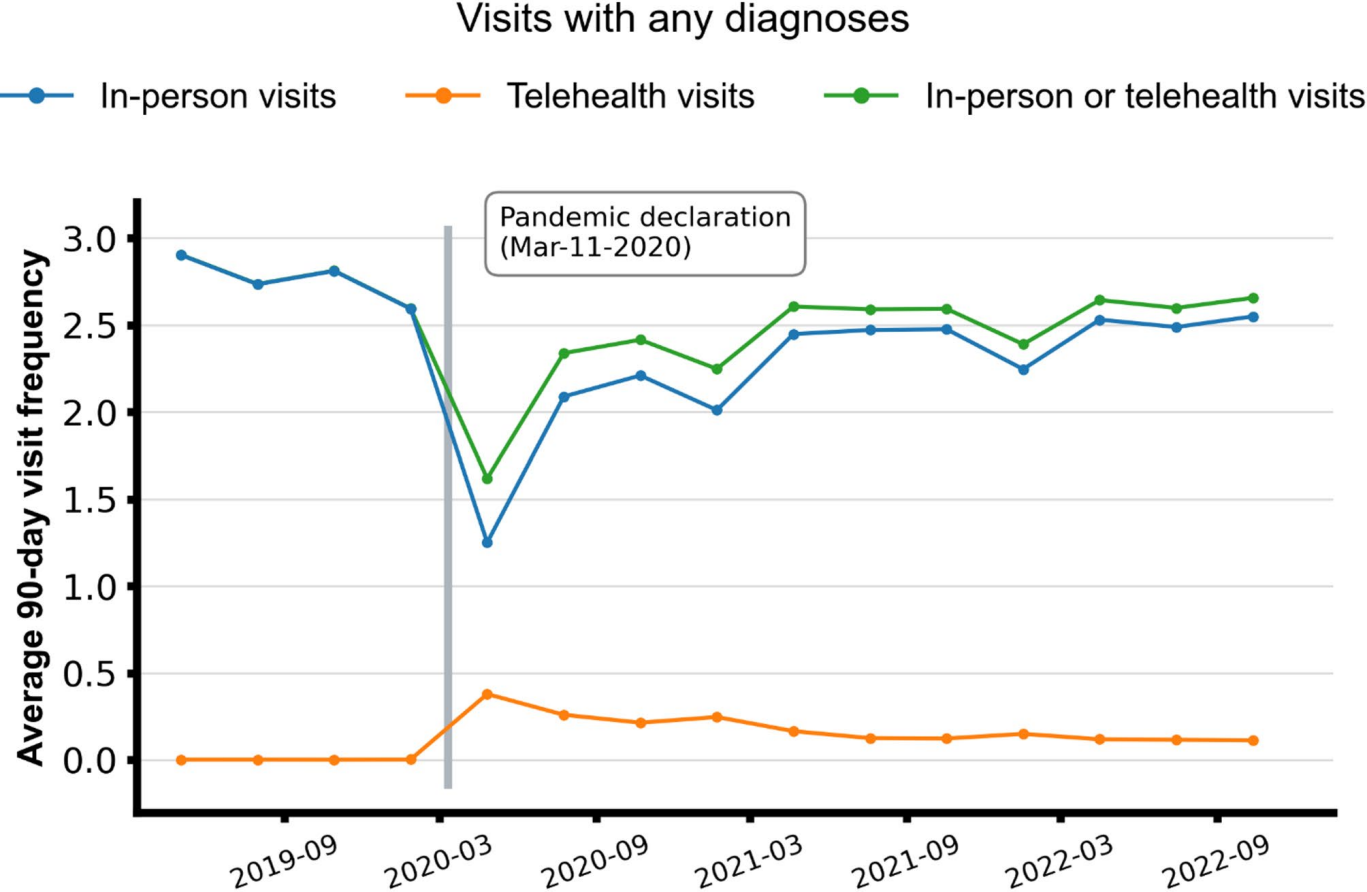
Descriptive trends of the 90-day rates of in-person and telehealth visits, overall population

For each 90-day interval during follow-up, we measured the number of outpatient visits, including in-person and telehealth, based on codes and modifiers listed in Supplemental Table 1. We compared the mean 90-day rates of in-person, telehealth, and total outpatient visits across three time periods: the baseline period (March 2019–March 2020), the pandemic period (March 2020–December 2021), and the post-pandemic period (December 2021–December 2022). We defined March 2020 as the beginning of the pandemic period because it coincided with the declaration of COVID-19 as a global pandemic and the start of national quarantine measures that markedly affected healthcare utilization. We defined December 2021 as the start of the post pandemic period because it marked the beginning of the first 90-day period after the lifting of travel restrictions [14]. This timing captures the period when outpatient care patterns began to stabilize, including potential shifts between telehealth and in-person utilization, and is consistent with prior literature assessing healthcare recovery following the COVID-19 pandemic [15, 16]. Analyses were performed for the overall sample as well as for quartiles of patients defined based on the average number of in-person visits during the baseline period, which consisted of four consecutive 90-day intervals. For the overall population and for each quartile group, we estimated the difference in mean 90-day total visits, in-person visits and telehealth visits interval during the pandemic and post-pandemic periods, as compared to the baseline. We also examined visits with a primary diagnosis of AF. Data analysis was performed using SAS, version 9.4 (SAS Institute). The study was approved by the Institutional Review Board at the University of California, San Diego as exempt as it was based on de-identified data.

## Results

The study included 124,483 Medicare beneficiaries with established AF (mean age 78.5 ± 9.0 years, 54.0% female, 90.0% non-Hispanic White) [8]. We identified four subgroups according to the quartiles in number of in-person visits during the baseline period with mean 90-day visits of 0.4 (group 1), 1.8 (group 2), 3.0 (group 3), and 5.8 (group 4).

Group 1 patients were the oldest (mean age 80 ± 9.8 years) and included the highest proportion of females (57.1%), compared with other groups, which had an average age of 78 years (sd 8.5) and approximately 53% females. The proportion of patients residing in urban areas increased from Group 1 (77.7%) to Group 4 (81.3%). Regarding clinical characteristics, Group 4 had the highest proportion of patients at high thromboembolic risk based on CHA₂DS₂-VASc scores (78%) and at high bleeding risk based on HAS-BLED scores (13%). In terms of socioeconomic factors, Group 1 included the greatest proportion of patients with dual Medicaid eligibility (24%) and receipt of low-income subsidy (26%), whereas Groups 2 to 4 had comparable proportions of patients with Medicaid dual eligibility (14%) and low-income subsidy (17%).

### Trends in total outpatient visits

The mean total outpatient visits (in-person + telehealth) significantly declined from the pre-pandemic [2.76 (95% CI, 2.63–2.89)] to the pandemic period [2.34 (95% CI, 2.08–2.60)] (Fig. 1). While telehealth visits increased during this period, they did not fully compensate for the reduction in in-person visits, leading to an overall decline in outpatient care utilization.

In the post-pandemic period, the rates of in-person visits increased from 2.14 (95% CI 1.81–2.46) to 2.45 (95% CI 2.32,2.59) but remained below pre-pandemic levels. However, after factoring in telehealth visits, total outpatient visits in the post-pandemic period [2.57 (95% CI, 2.44–2.69)], reaching levels comparable to those observed before the pandemic.

### Differences in outpatient utilization across subgroups

**Fig. 2:**
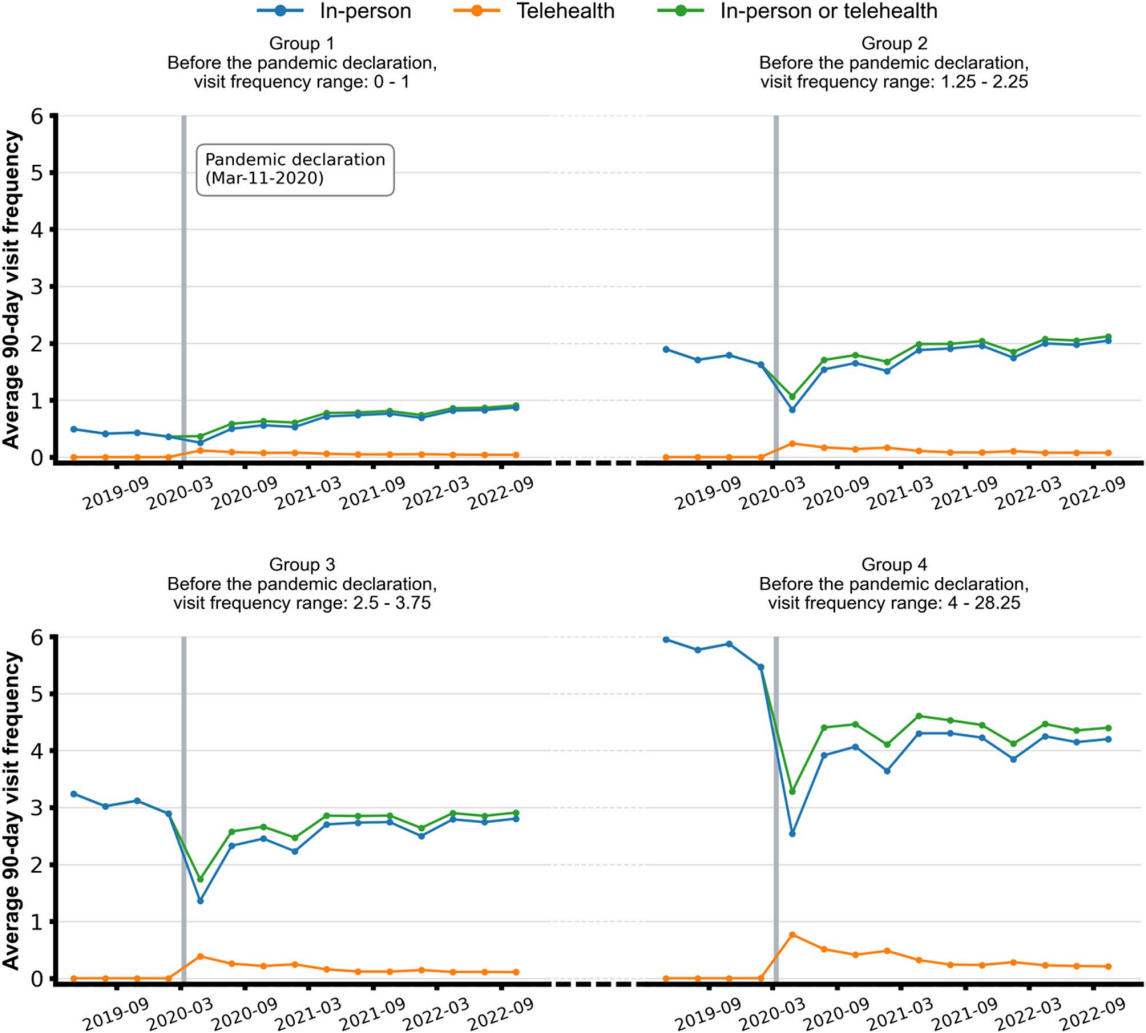
Descriptive trends of 90-day in-person and telehealth visits, by quartile groups

There were important differences in the trends of total visits across subgroups (Fig. 2). For Group 1, the subgroup with the lowest utilization at baseline, the mean 90-day total visit rate increased from 0.42 (95% CI, 0.37–0.48) to 0.65 (95% CI, 0.53–0.76). In contrast, total visits declined significantly for Groups 3 and 4 (Table 1).

**Table 1:** Average number of 90-day in-person and telehealth visits.

	Average number of visits per 90-day interval (95% confidence interval)			Direction of change	
	Baseline period	Pandemic period	Post-pandemic period	Pandemic vs. baseline	Post-pandemic vs. baseline
	(March 2019 - 2020)	(March 2020 - Dec 2021)	(Dec 2021 - 2022)		
All visits					
Overall Population	2.76 (2.63,2.89)	2.34 (2.08,2.60)	2.57 (2.44,2.69)	↓	↓Partial recovery
Group 1	0.42 (0.37,0.48)	0.65 (0.53,0.76)	0.84 (0.77,0.92)	↑	↑
Group 2	1.76 (1.64,1.87)	1.75 (1.50,2.00)	2.02 (1.90,2.13)	≈No change	↑
Group 3	3.07 (2.92,3.21)	2.57 (2.28,2.88)	2.82 (2.70,2.95)	↓	↓
Group 4	5.77 (5.56,5.97)	4.26 (3.92,4.60)	4.34 (4.19,4.48)	↓	↓
In-person visits					
Overall Population	2.75 (2.63,2.89)	2.14 (1.81,2.46)	2.45 (2.32,2.59)	↓	↓Partial recovery
Group 1	0.42 (0.37,0.48)	0.58 (0.44,0.71)	0.80 (0.72,0.88)	↑	↑
Group 2	1.75 (1.64, 1.87)	1.61 (1.32,1.90)	1.94 (1.80, 2.07)	≈ No change	↑
Group 3	3.07 (2.92,3.21)	2.36 (2.00,2.72)	2.71 (2.57,2.85)	↓	↓
Group 4	5.76 (5.56, 5.97)	3.86 (3.39,4.32)	4.11 (3.93,4.29)	↓	↓
Telehealth visits					
Overall Population	0.00 (0.00,0.00)	0.22 (0.14,0.28)	0.12 (0.11,0.14)	↑	↑
Group 1	0.00 (0.00,0.00)	0.07 (0.05,0.10)	0.04 (0.03,0.04)	↑	↑
Group 2	0.00 (0.00,0.00)	0.14 (0.10,0.18)	0.08 (0.07,0.10)	↑	↑
Group 3	0.00 (0.00,0.00)	0.22 (0.14,0.28)	0.12 (0.10,0.13)	↑	↑
Group 4	0.00 (0.00,0.00)	0.43 (0.29,0.56)	0.24 (0.20,0.26)	↑	↑

Patients were categorized into four groups (Quartiles 1–4) according to the number of in-person outpatient visits they had during the baseline period (March 2019 – March 2020). Group 1 represented patients with the lowest baseline outpatient utilization, whereas Group 4 represented those with the highest pre-pandemic utilization levels

Arrows (↑/↓) indicate the direction of change relative to baseline. Groups 1 and 2 showed increased utilization in the post-pandemic period compared to baseline period, while Groups 3 and 4 experienced persistent declines in total and in-person visits despite modest telehealth uptake following the onset of the pandemic

In the post-pandemic period, mean 90-day total visits for Groups 1 and 2 increased compared to the baseline level, driven by increases in both telehealth and in-person visits. In contrast, Groups 3 and 4 continued to experience a decline in total visits compared to pre-pandemic levels, with Group 3 decreasing from 3.07 (95% CI, 2.92–3.21) to 2.82 (95% CI, 2.70–2.95) and Group 4 from 5.77 (95% CI, 5.56–5.97) to 4.34 (95% CI, 4.19–4.48).

Telehealth use increased during the pandemic, accounting for approximately 8–11% of all outpatient visits, and then declined to 4–6% in the post-pandemic period (Table 2). While telehealth visits increased for these groups, they were insufficient to compensate for the continued reduction in in-person visits (Fig. 2).

**Table 2:** Telehealth outpatient visits per 90-day interval and proportion of telehealth visits

Telehealth outpatient visits per 90-day interval and proportion of telehealth visits				
	Baseline Period	Pandemic Period	Post-pandemic Period	
	(March 2019 – 2020)	(March 2020 – Dec 2021)	(Dec 2021 – 2022)	
Overall Population	0 (0)	0.22(9%)	0.12 (5%)	
Group 1	1 (0)	0.07(11%)	0.04 (5%)	
Group 2	2 (0)	0.14(8%)	0.08 (4%)	
Group 3	3 (0)	0.22(9%)	0.12 (4%)	
Group 4	4 (0)	0.43(10%)	0.24 (6%)	

The proportion of telehealth visits was calculated as (telehealth visits/ total outpatient visits) × 100

Across all groups, telehealth visits accounted for approximately 8–11% of total outpatient visits during the pandemic, declining to 4–6% in the post-pandemic period

The differences in visits with a primary diagnosis of AF were consistent with the overall visit patterns (Supplemental Fig. 2). Telehealth visits increased for all groups, while in-person visits decreased following the onset of the pandemic. In the post-pandemic period, Groups 3 and 4 continued to exhibit lower levels of utilization compared with the pre-pandemic baseline.

## Discussion

Using a nationally representative sample of Medicare beneficiaries with established AF, telemedicine use coupled with the recovery in the rates of in-person visits after December 2021 resulted in a return to the levels of outpatient visits observed before the COVID-19 pandemic. Pandemic-related changes in the frequency of outpatient visits differed substantially across subgroups defined by baseline health care utilization. Patients with lower rates of visits at baseline experienced an increase in health care utilization during the pandemic, while patients with higher baseline outpatient care utilization saw a reduction in the number of outpatient visits, even after accounting for increased use of telehealth.

Our study contributes to the growing body of literature on pandemic disruptions in outpatient care [17, 18]. We observed that, in the post-pandemic period, the rates of in-person visits were still significantly lower than the pre-pandemic levels; however, after accounting for telemedicine visits, the total number of visits in the post pandemic period were comparable to those observed before March 2020. Despite the role of telemedicine in closing the gap in care compared with pre-pandemic levels, the overall rates of telemedicine visits were modest, with telehealth visits representing an average of only approximately 10% of total outpatient encounters, even during the peak of the pandemic. On average, telehealth visits increased by 0.12 visits per 90-day period across subgroups, indicating small absolute changes in utilization. Given the modest magnitude of these changes, the findings likely reflect population-level adjustments in healthcare access rather than clinically meaningful shifts in AF management at the individual patient level.We found significant heterogeneity in health care utilization patterns based on outpatient care utilization at baseline, defined as the number of visits per 90 days in the year before the pandemic. Specifically, for patients with lower baseline outpatient visits, there was an increase in the number of total visits during the pandemic and post-pandemic periods, driven by increases in both telehealth and in-person visits. In contrast, patients with higher baseline outpatient care utilization presented levels of care utilization below those observed in the pre-pandemic period. These patterns could partially be explained by changes in the underlying health status of subgroups over time. For instance, it is possible that patients in groups 3 and 4, which presented high levels of utilization in the pre-pandemic period, experienced decreases in outpatient visits because some of the primary concerns driving healthcare utilization at baseline were resolved. Conversely, patients in Groups 1 and 2 may have experienced disease progression, leading to an increase in their healthcare utilization over time. This is a limitation of our analysis, which classified patients on time-fixed subgroups, based on the levels of outpatient visits in the baseline period only. Future studies should examine whether the observed utilization changes reflect differences in patient health status or led to changes in AF control. In addition, we did not examine visit-level or system-level characteristics, such as specific service categories or provider availability, which could further clarify the reasons for the observed heterogeneity across groups with different levels of healthcare utilization. This study focused on describing population-level trends in outpatient and telehealth utilization among patients with AF. Future research could apply statistical modeling approaches, such as mixed-effects models, to examine associations between utilization patterns and clinical, provider, or system-level determinants of care recovery.

## Conclusion

Telehealth use and the gradual recovery of in-person visits in the post-pandemic period contributed to the restoration of outpatient care levels across the population of patients with AF.

However, differences heterogeneity across subgroups suggest that recovery of outpatient care was not uniform across patients, with marked differences based on patient’s levels of baseline outpatient utilization pre-pandemic utilization.

## Limitations

Our study is subject to two additional limitations. First, the study did not examine the chief compliant for outpatient visits, as our objective was to assess overall healthcare utilization. This limits our ability to determine whether changes in visit frequency were explained by changes in the trend of specific conditions. Second, we were unable to evaluate whether changes in visit levels were driven by variation in provider availability, or by changes in patient health care seeking behavior.

## Supplementary Information

Below is the link to the electronic supplementary material.

Supplementary Material 1

Supplementary Material 2

## Data Availability

The data used in this study are not publicly available due to restrictions outlined in the Centers for Medicare & Medicaid Services (CMS) Data Use Agreement. The findings supporting the conclusions of this study may be available from the corresponding author upon reasonable request and subject to CMS approval.
