# Supplementary material for: Trends in outpatient care utilization for patients with established atrial fibrillation before and after the Covid-19 pandemic: a nationwide analysis of claims data
Source: BMC Res Notes. 2025 Nov 27;18:499. doi: 10.1186/s13104-025-07569-6 (PMC12661868; doi:10.1186/s13104-025-07569-6)
Supplement: Supplementary file 1 — Supplementary Material 1 [file 13104_2025_7569_MOESM1_ESM.docx]

**Supplements**

**Trends in Outpatient Care Utilization for Patients with Established Atrial Fibrillation Before and After the Covid-19 Pandemic: A Nationwide Analysis of Claims Data**

Lanting Yang, PhD, MPH, Shangbin Tang, MS, Jingchuan Guo, MD, PhD, Nico Gabriel, MA, A. Mark Fendrick, MD, Nimish Patel, PharmD, PhD, Utibe R. Essien, MD, MPH , Jared W. Magnani, MD, MS, Walid F Gellad, MD, MPH, Inmaculada Hernandez, PharmD, PhD

Supplemental Figure 1 Overview of the Sample Selection.

Supplemental Table 1 Definitions of Telehealth and In-person Visits.

Supplemental Table 2 Baseline Patient Characteristics, by Quartile Group

Supplemental Figure 1 Overview of the Sample Selection.


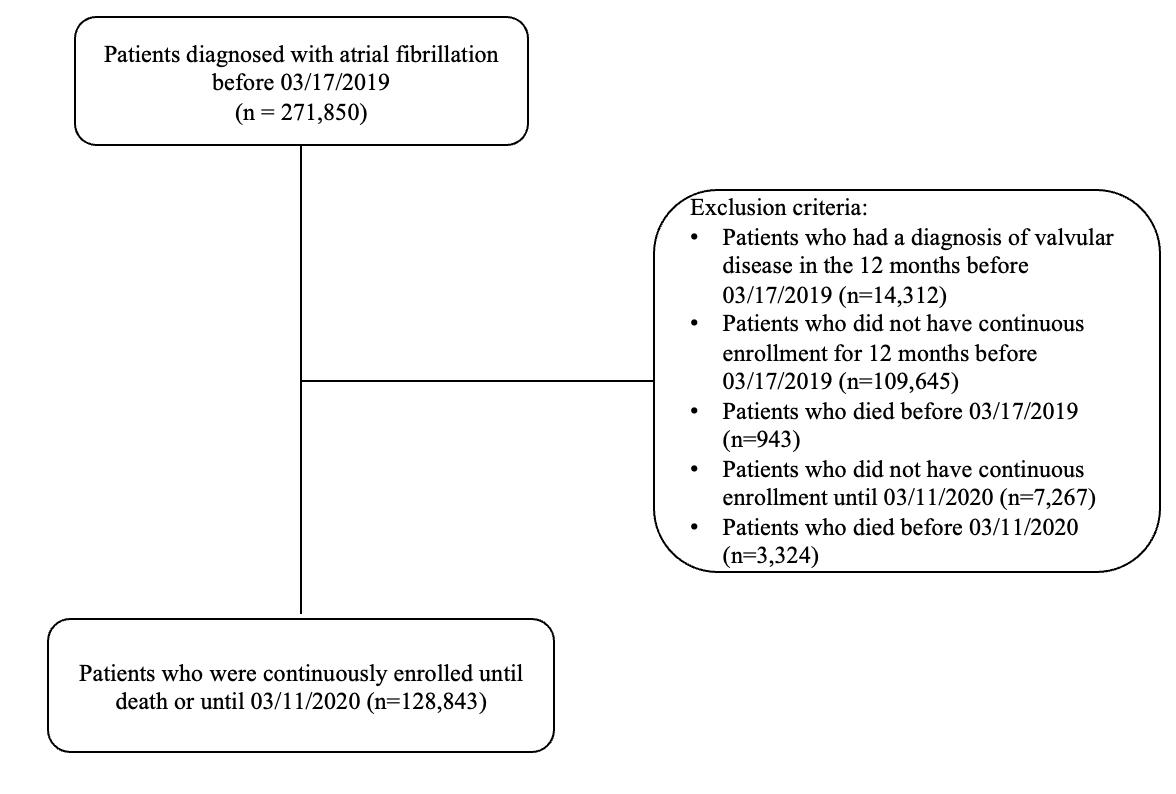


Supplemental Table 1 Definitions of Telehealth and In-person Visits.

| **Visit Type** | **Current Procedural Terminology Codes** | **Modifier** |
| --- | --- | --- |
| Telehealth outpatient visits | G0425-G0427 | None |
|  | 99201-99215 Office or Other Outpatient Services | 95 or GT |
|  | 99334-99337 Home and Domiciliary Visits |  |
|  | 99341-99345 Home or Residence Services- New patient |  |
|  | 99347-99350 Home or Residence Services- Established patient |  |
|  | G0438, G0439 Annual wellness visits |  |
|  | G0402 Initial preventive physical examination |  |
| In-person outpatient visits | 99201-99215 Office or Other Outpatient Services | Without 95 or GT |
|  | 99381-99387 Preventive Medicine Services- New Patient Visit |  |
|  | 99391-99397 Preventive Medicine Services- Established Patient |  |
|  | 99417 Prolonged outpatient evaluation and management services |  |
|  | G0438, G0439 Annual wellness visits |  |

Supplemental Table 2 Baseline Patient Characteristics, by Quartile Group

| **Patient characteristics, No (%)** | **Group 1  (N=29748 )** | **Group 2  (N=32895)** | **Group 3  (N=30824)** | **Group 4 (N=31016)** |
| --- | --- | --- | --- | --- |
| **Demographics** |  |  |  |  |
| Age, Mean(Std.) | 80(9.8) | 78(9.0) | 78(8.6) | 78(8.5) |
| Female | 16994(57.1) | 17543(53.3) | 16199(52.6) | 16371(52.8) |
| Race |  |  |  |  |
| Non-Hispanic White | 26348(88.6) | 29620(90.4) | 27985(90.8) | 28165(90.8) |
| Non-Hispanic Black | 1964(6.6) | 1567(4.8) | 1327(4.3) | 1335(4.3) |
| Hispanic | 331(1.1) | 300(0.9) | 313(1.0) | 345(1.1) |
| Other | 1105(3.7) | 1408(4.3) | 1199(3.9) | 1171(3.8) |
| Urbanicity |  |  |  |  |
| Urban areas | 23096(77.7) | 25020(76.1) | 23784(77.2) | 25198(81.3) |
| Rural areas | 6634(22.3) | 7862(23.9) | 7013(22.8) | 5786(18.7) |
| **Clinical Characteristics** |  |  |  |  |
| CHA2DS2-VASc Score |  |  |  |  |
| Low Risk | 1075(3.6) | 1186(3.6) | 745(2.4) | 437(1.4) |
| Moderate Risk | 2839(19.6) | 7882(24.0) | 6983(22.7) | 6355(20.5) |
| High Risk | 22844(76.8) | 23827(72.4) | 23096(75.0) | 24224(78.1) |
| HAS-BLED Score |  |  |  |  |
| 0 | 1722(5.8) | 1654(5.0) | 1010(3.3) | 549(1.8) |
| 1-2 | 26653(89.6) | 29254(88.9) | 27133(88.0) | 26406(85.1) |
| >=3 | 1373(4.6) | 1987(6.0) | 2681(8.7) | 4061(13.1) |
| Use of oral anticoagulation |  |  |  |  |
| Warfarin | 4298(14.5) | 5242(15.9) | 5249(17.0) | 6211(20.3) |
| ACDOAC | 9172(30.8) | 11500(35.0) | 11163(36.2) | 10810(34.9) |
| No use | 16278(54.7) | 16153(49.1) | 14412(46.8) | 13995(45.1) |
| **Sociodemographic** |  |  |  |  |
| Medicaid dual eligibility | 7116(23.9) | 4850(14.7) | 4142(13.4) | 4620(14.9) |
| Receipt of low-income subsidy | 7851(26.4) | 5768(17.5) | 4960(16.1) | 5408(17.4) |
